# Supplementary material for: The Excited State Dynamics of a Mutagenic Cytidine Etheno Adduct Investigated by Combining Time-Resolved Spectroscopy and Quantum Mechanical Calculations
Source: J Phys Chem Lett. 2021 Dec 30;13(1):251–7. doi: 10.1021/acs.jpclett.1c03534 (PMC9135321; doi:10.1021/acs.jpclett.1c03534)
Supplement: Supplementary file 1 — jz1c03534_si_001.pdf [file jz1c03534_si_001.pdf]

Supplementary Information

The Excited State Dynamics of a Mutagenic Cytidine Etheno Adduct Investigated by Combining Time-Resolved Spectroscopy and Quantum Mechanical Calculations

Paloma Lizondo-Aranda[a], Lara Martínez-Fernández[b], Miguel A. Miranda[a], Roberto Improta[c,*], Thomas Gustavsson[d,*] and Virginie Lhiaubet-Vallet[a,*]

[a]*Instituto Universitario Mixto de Tecnología Química (UPV-CSIC), Universitat Politècnica de Valencia, Consejo Superior de Investigaciones Científicas, Avda de los Naranjos s/n, 46022 Valencia, Spain.*
[b]*Departamento de Química, Facultad de Ciencias and IADCHEM (Institute for Advanced Research in Chemistry) Universidad Autónoma de Madrid, Cantoblanco, 28049 Madrid, Spain*
[c]*Istituto di Biostrutture e Bioimmagini, CNR, Via Mezzocannone 16, I-80134 Napoli, Italy.* [d]*Université Paris-Saclay, CEA, CNRS, LIDYL, 91191 Gif-sur-Yvette, France.*

Index

Experimental and computational procedures - *Pages S2-S3*

Figure S1. Absorption spectra of 2′-deoxycytidine in PBS pH 7.4 and in citric acid buffer pH 3 – *Page S4*

Figure S2. Steady-state fluorescence spectra of εdC in PBS 0.1M pH 7.4 obtained at different excitation wavelengths - *Page S5*

Figure S3. Computational model adopted to study εC in water solution at neutral (εC•2H_2O left) and acidic (εCH^+•2H_2O right) pH - *Page S6*

Figure S4. Natural Transition Orbital (NTO) associated to the three lowest energy excited states in εC and εC^+•2H_2O in water, according to PCM/M052X/6-31G(d) calculations - *Page S7*

Figure S5. Schematic description of the proposed decay mechanism for εC and εCH^+ in gas phase according to CASPT2/CASSCF calculations - *Page S8*

Figure S6. Total fluorescence decays obtained from a solution of εdC in PBS pH 7.4 at different emission wavelengths after excitation at λ_{exc}= 267 nm - *Page S9*

Figure S7. Selected anisotropy decays obtained from a solution of εdC in PBS pH 7.4 at different emission wavelengths after excitation at λ_{exc}= 267 nm - *Page S10*

Figure S8. Fluorescence emission of εdC at pH 7.4 at different delays after the pump. (A) up to 0.3 ps, (B) from 0.3 to 2.7 ps - *Page S11*

Figure S9. Total fluorescence decays obtained for εdCH^+ in PBS pH 3 at different emission wavelengths after excitation at λ_{exc}= 267 nm - *Page S12*

Figure S10. Fluorescence emission of εdCH^+ at pH 3 at different delays after the pump. (A) up to 0.1 ps, (B) from 0.1 to 2.6 ps, (C) at longer timescale from 0 to 29 ps - *Page S13*

Figure S11. Selected time-resolved fluorescence spectra of εdC in PBS at pH 7.4 (black dots) at different delay times after excitation at λ_{exc}= 267 nm - *Page S14*

Figure S12. Time evolution of (A) total intensity, (B) peak frequency and (C) spectral width using log-normal functions - *Page S15*

Figure S13. Selected time-resolved fluorescence spectra of εdC in PBS pH 3 (black dots) at different delay times after excitation at λ_{exc}= 267 nm - *Page S16*

Figure S14. Time evolution of (A) total intensity, (B) peak frequency and (C) spectral width using log-normal functions at a time window of 3 ps - *Page S17*

Figure S15. Time evolution of (A) total intensity, (B) peak frequency and (C) spectral width using log-normal functions at a time window of 30 ps - *Page S18*

Figure S16. CASSCF Active Space for εC calculations – *Page S19*

Figure S17. CASSCF Active Space for εCH^+ calculations – *Page S19*

Table S1. Optical properties of εdC and dC in PBS 0.1 M at pH 7.4 and 3 - *Page S20*

Table S2. Vertical absorption and emission energies (in eV) computed for εC at different TD-DFT levels of theory - *Page S21*

Table S3. Vertical absorption and emission energies (in eV) computed for εCH^+ at different TD-DFT levels of theory - *Page S22*

Table S4. Vertical absorption energies (in eV) computed for εC and εCH^+ in the gas phase. MSCASPT2 calculations. Oscillator strength is given in parentheses - *Page S23*

Table S5. Fitted parameters for the decays of εdC in PBS. The average lifetime was calculated as $<\tau> = a_1\tau_1 + a_2\tau_2$. The anisotropy decay time $\tau_{R,2}$ was fixed to 55 ps - *Page S24*

Table S6. Fitted parameters for the decays of εdCH^+ at pH 3 – *Page S25*

References – *Pages S26-S27*

Experimental procedures

Reagents and Solvents. 3,N4-etheno-2'-deoxycytidine (εdC), sodium phosphate monobasic (Na_2HPO_4), sodium phosphate dibasic (Na_2HPO_4), sodium citrate dihydrate, citric acid, methanol, diethyl ether and acetonitrile were purchased from Carbosynth and Sigma-Aldrich and used as received. All the solvents were spectroscopic grade and buffers were made following the AAT Bioquest buffer protocol.

UV-Vis measurements. Absorption spectra were recorded in a Perkin Lambda 850 spectrophotometer, using quartz cuvette with optical path of 1 cm.

Steady state fluorescence. Spectra were measured upon 267 nm excitation with a Fluorolog-3 (Horiba, Jobin-Yvon) fluorimeter. Solutions of εdC or TMP (used as standard) with an absorbance of 0.1 at the excitation wavelength were prepared. As the emissions of the neat buffered solutions were not negligible compared to that of εdC, they were recorded separately and subtracted from the spectra of the sample to remove their contribution. Moreover, the recorded spectra were corrected for the response of the detection system. Quantum yield was determined using thymidine monophosphate as reference ($\phi_F = 1.54 \times 10^{-4}$). [1]

Upconversion technique. A detailed description of the femtosecond fluorescence upconversion equipment has been given earlier. [2] Briefly, the 267 nm excitation was generated as a third harmonic of a femtosecond mode-locked Ti-sapphire laser (Coherent MIRA 900). The average excitation power was set at 50 mW. The εdC solution (30 mL, 0.5 OD at 267 nm) was circulated in a flow system with a moving 1 mm flow-cell in order to avoid degradation.

The fluorescence from the cell was collected and focused into a 1 mm BBO type I crystal using two off-axes parabolic mirrors. It was further passed through a cutoff filter (WG305 or WG320) in order to eliminate the weak remaining UV excitation light.

After the mixing of the fluorescence and the IR gating pulse in the NL crystal, the generated sum-frequency light was focused on the entrance slit of a double monochromator equipped with a photomultiplier (Hamamatsu 1527P) connected to a photon counter (Stanford SR400). Fluorescence decays were recorded at selected wavelengths with parallel (I_{par}(t)) and perpendicular (I_{perp}(t)) excitation/detection configurations. These were realized by controlling the polarization of the exciting beam with a zero-order half wave plate. The temporal scans were repeated several times and then averaged in order to improve the statistics. For visualization, total fluorescence decay kinetics (F(*t*)) were constructed from the parallel and perpendicular signals according to the equation: $F(t) = I_{par}(t) + 2\, I_{perp}(t)$. [3]

Likewise, the fluorescence anisotropy r(t) was constructed according to the equation: $F(t) = (I_{par}(t) - I_{perp}(t))/F(t)$.

For the data treatment, parallel and perpendicular scans were treated together, allowing both the intensity and the anisotropy decays to be model-fitted simultaneously.

To this end, we performed a merged nonlinear fitting/deconvolution process using the impulse response model functions

$i_{par}(t) = (1 + 2r(t))f(t)$

$i_{perp}(t) = (1 - r(t))f(t)$

convoluted by the Gaussian instrument response function (irf) $G(t)$: $i(t) \otimes G(t)$. The fwhm value of the Gaussian irf was about 400 fs.

The model functions thus obtained were fitted to the experimental parallel (I_{par}) and perpendicular (I_{perp}) signals. Mono- or biexponential functions were used for $f(t)$, while $r(t)$ was taken as monoexponential. The average lifetime $<\tau>$ was determined from fitted parameters using the equation: $<\tau> = a_1\tau_1 + a_2\tau_2$.

Time resolved fluorescence spectra were recorded at magic angle between 300 and 385 nm by scanning the monochromator with a step of 5 nm. The detection system is blind around 400 nm due to scattered excitation light and the red-wing of the spectrum was too weak to record separately. About 30 time-resolved spectra equally spaced in time were recorded over a time window of 3 or 30 ps. The full wavelength – time sampling was repeated several times and then averaged in order to improve the statistics.

Resulting spectra were corrected with regards to the spectral sensitivity of the system using a phenomenological correction curve obtained by comparing a reference FU spectrum to the corresponding steady-state spectrum. For the pH 7.4 recordings, the long-time spectra (> 200 ps) of the dye DMQ were used (external calibration), while for the pH 3 recordings, the long-time spectra (< 20 ps) of εdC itself were used (internal calibration).

Computational details.

Water solution. In order to study the behavior of εdC and $εdCH^+$ in water we have adopted the computational models depicted in Figure S3 and denoted εC and $εCH^+$, respectively, where the sugar is mimicked by a methyl group, bulk solvent effects are included with the polarizable continuum (PCM) model,[4] and the effect of solute-solvent hydrogen bond interactions considered by explicitly including two H_2O molecules, with the arrangement shown the Figure. Our previous study on cytosine shows indeed that substitution of the sugar with methyl group does not have a significant impact on the excited state behaviour.[5] Calculations have been also performed without including explicit water molecules. DFT and TD-DFT, using the M052X functional,[6,7] have been used as reference computational model, since they have been already profitably adopted to study the photoactivated behavior of cystosine derivatives in solution.[5,8] All the TD-DFT calculations have been performed by the Gaussian16 program.[9]

Gas Phase. In parallel we have mapped the PES of εCH and $εCH^+$ in the gas phase by Complete Active Space Self Consistent Field (CASSCF)[10,11] optimization of minima and state crossings and minimum energy path calculations. Energies have been corrected using second-order perturbation theory (CASPT2) in its single and multistate (MS)[12] formulation with an IPEA shift[13] of 0.0 a.u. and an imaginary level shift[14] of 0.3 a.u. The selected active space (Figure S16), 16 electrons in 12 orbitals, contains all the π and π^* orbitals together with the Lone Pairs of the O and N8 atoms for εCH. Since the latter is not present in $εCH^+$ the active space is reduced to 14 electrons in 11 orbitals. An equal weight state-average over 5 states was used throughout (Figure S17). To be consistent with the DFT calculations these calculations were also done using the 6-31G(d) basis set OpenMolcas software has been used for these calculations.[15]

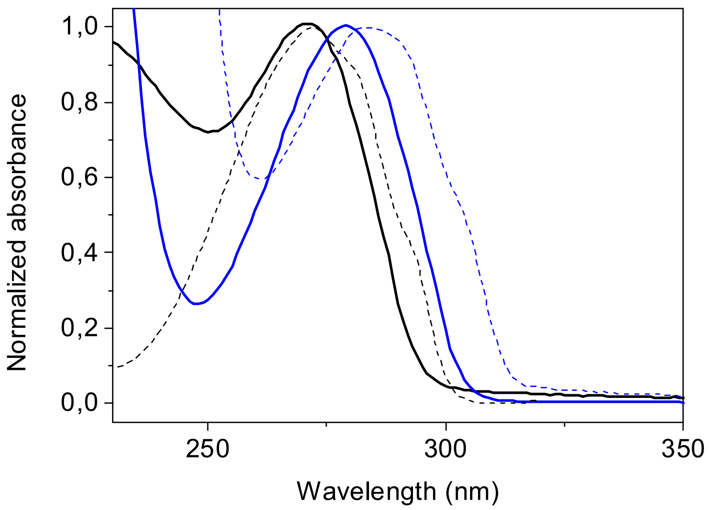

Figure S1. Absorption spectra of 2´-deoxycytidine in PBS pH 7.4 (black line) and in citric acid buffer pH 3 (blue line). For comparison, the absorption spectra of εdC under the same pH conditions are represented with dashed lines.

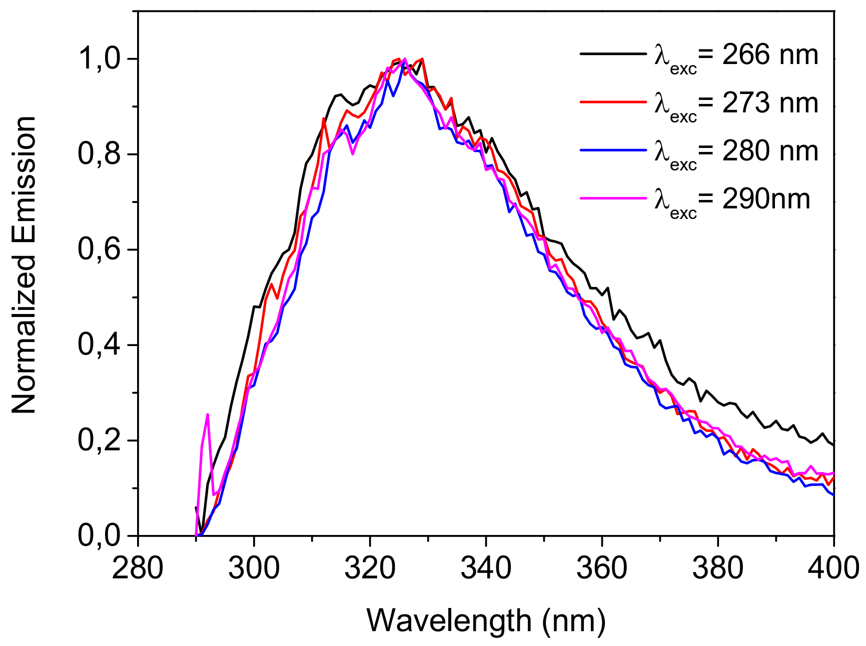

Figure S2. Steady-state fluorescence spectra of εdC in PBS 0.1M pH 7.4 obtained at different excitation wavelengths.

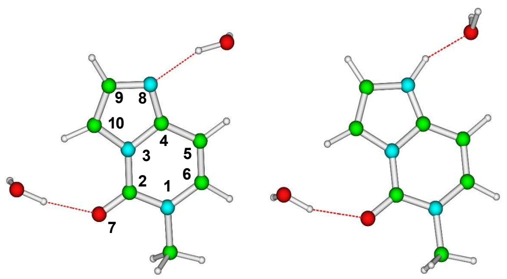

Figure S3. Computational model adopted to study εC in water solution at neutral (εC•2H_2O left) and acidic (εCH^+•2H_2O right) pH.

Section A. Franck-Condon Region

The natural transition orbitals (NTO)[16] associated to the $S_0 \rightarrow S_1$ and $S_0 \rightarrow S_2$ electronic transitions εC•2H_2O and εCH^+•2H_2O (Figure S3) are reported in Figure S4. NTOs is a useful tool to describe the electronic transitions in terms of one-particle excitations.

The lowest energy excited states energies and oscillator strengths at the FC region are collected in Table S2 and S3. The S_1 can be described as bright ππ* excitation (with a prevalent HOMO→LUMO contribution), hereafter ππ***1**, from an orbital with strong C5-C6 bonding character to an orbital which is antibonding with respect to this bond. On the other hand, there is a strong contribution of the 5-member ring atoms to this excitation. The second lowest energy state S_2 is also a bright ππ* state (hereafter denoted ππ***2**), with the strongest contribution coming from HOMO→LUMO+1 excitation. This picture is rather similar to that obtained for 1-methylcytosine (hereafter simply C) in water. On the other hand, as shown in Table S2, significant differences are found with respect to the canonical nucleobase. In addition to a weak red-shift of the lowest energy absorption band, we observe that in εC ππ***2** gets closer to ππ***1**, while its intensity decreases. Always keeping in mind that vibronic effects strongly affect the experimental lineshapes,[17] the contribution of ππ***2** could explain why the absorption maximum of εC (273 nm) is very similar to that of C (271 nm). Moreover, the large shoulder/shallow maximum at 240-250 nm, which is present in the experimental spectrum of C (see Figure S1) and it is due to ππ***2,** disappears. The increase of the molar absorption coefficient with respect to C is reproduced by our calculations.

The role of the dark states and the effect of protonation are discussed in the main text.

It is worth noting that the explicit inclusion of H_2O molecules has a limited impact on the absorption spectra (Table S2 and S3). Finally, the extension of the basis set leads to a moderate red-shift of the spectra of both εC and εCH^+, (Table S2 and S3) but does not change the qualitative picture obtained with the smaller 6-31G(d) basis set.

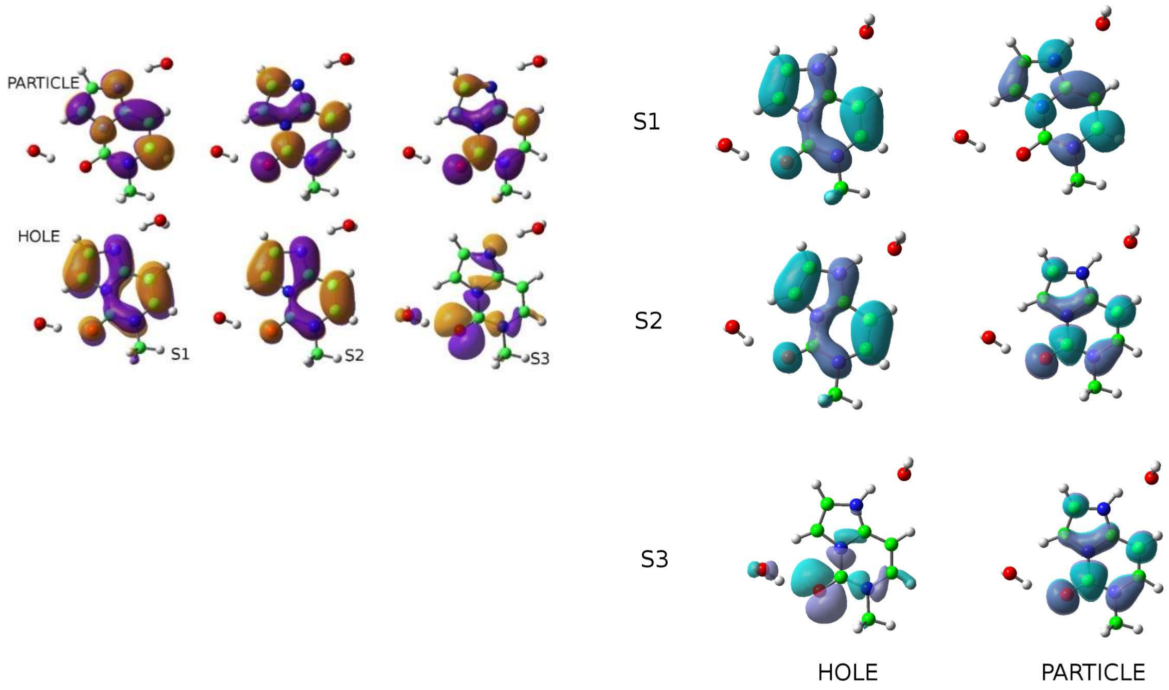

Figure S4. Natural Transition Orbital (NTO) associated to the three lowest energy excited states in εC•2H_2O (left) and (right) εC$^+$•2H_2O in water, according to PCM/M052X/6-31G(d) calculations.

Section B. S_1 minimum

S_1 geometry optimization leads to a stable minimum of the Potential Energy Surface (PES), denoted ππ*1-min, both for εC•2H_2O and εCH$^+$•2H_2O. The vertical emission energies obtained from these minima are given in Tables S2 and S3. Calculations reproduce the spectral red-shifts associated with protonation, being more important for absorption than for fluorescence (Figure 1 and Table S1). This is also well reflected, both experimentally and theoretically, by a higher Stokes (ν) shift under neutral (ν^{exp}=0.73 eV vs ν^{theo}=1 eV) conditions than at acidic pH (ν^{exp}=0.63 eV vs ν^{theo}=0.8 eV). The small overestimation of ν^{theo} can be interpreted in terms of vibronic effects which affect absorption and emission maxima in opposite directions.

Moreover, a more careful treatment of dynamical solvation effects could provide refined, and more reliable, values. The main structural changes with respect to the Franck Condon point involve the lengthening of the C5-C6 and C9-C10 double bonds. Interestingly the S_1 minimum keeps the planarity typical of the S_0 minimum, at difference with what happens for C, where a shallow minimum, with the ring adopting a strongly bent structure, is predicted.[5,8]

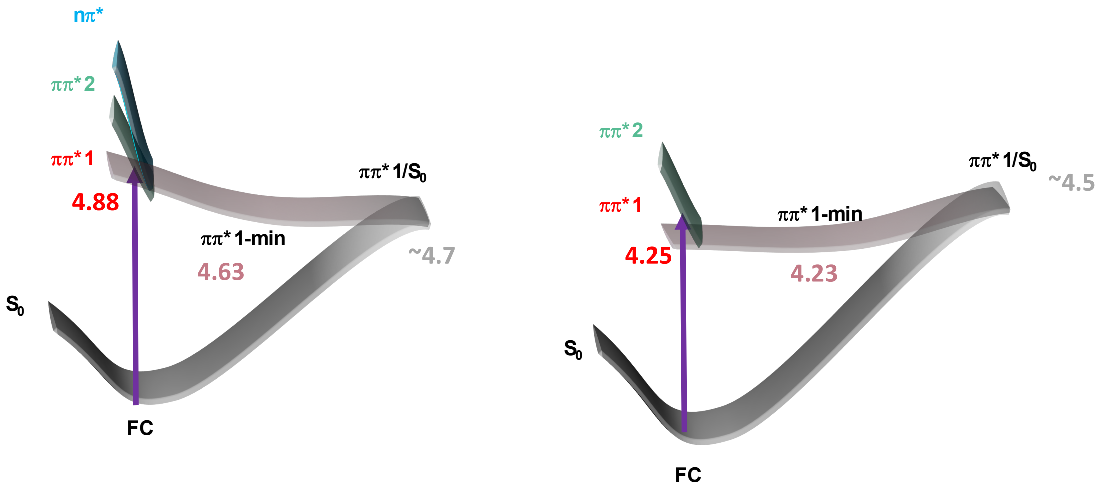

Figure S5. Schematic description of the proposed decay mechanism for εC (left) and $εCH^{+}$ (right) in gas phase according to CASPT2/CASSCF calculations.

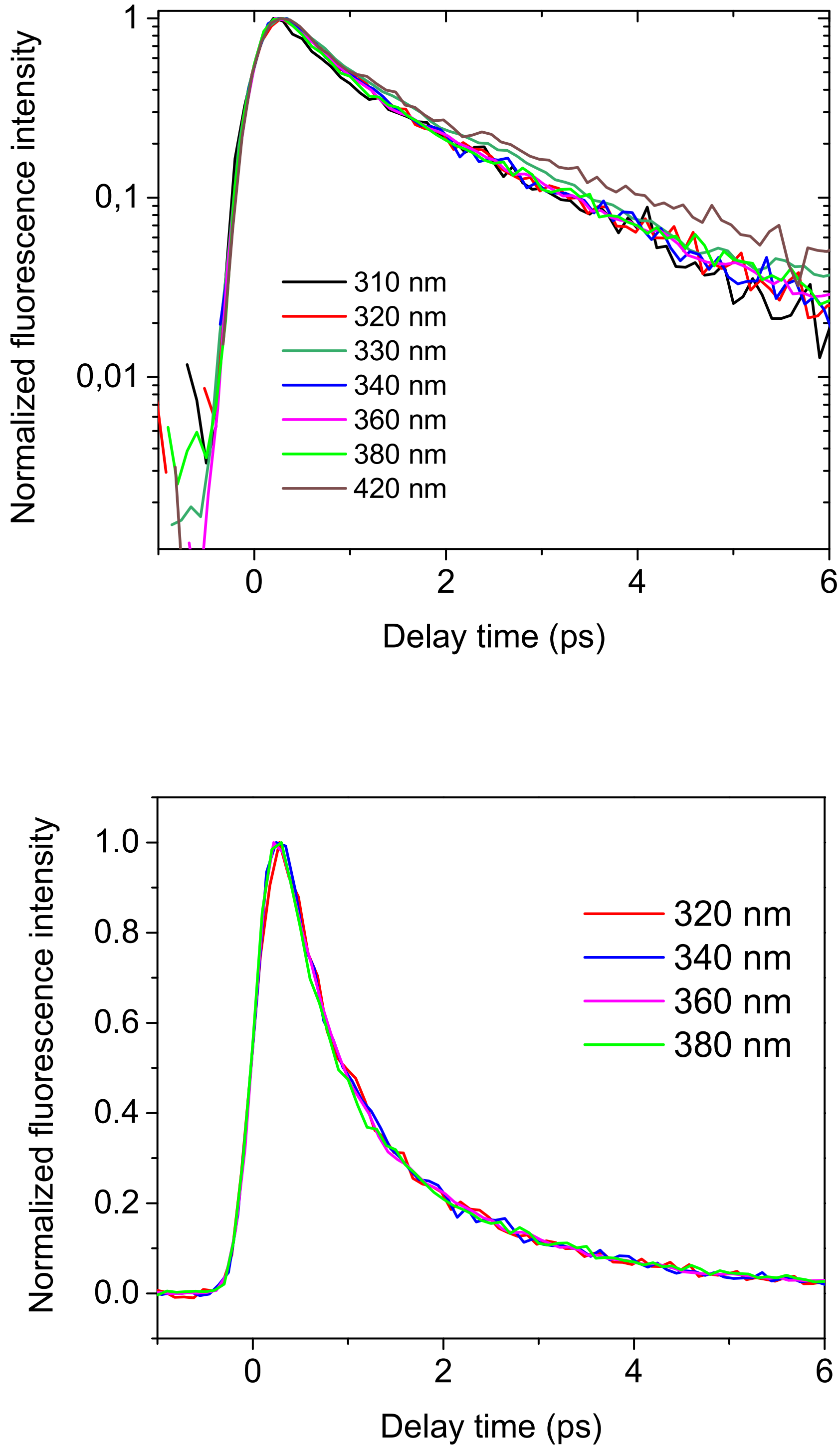

Figure S6. Total fluorescence decays, in semi-logarithmic (up) and lineal (bottom) scale, obtained from a solution of εdC in PBS pH 7.4 at different emission wavelengths after excitation at λ_{exc}= 267 nm.

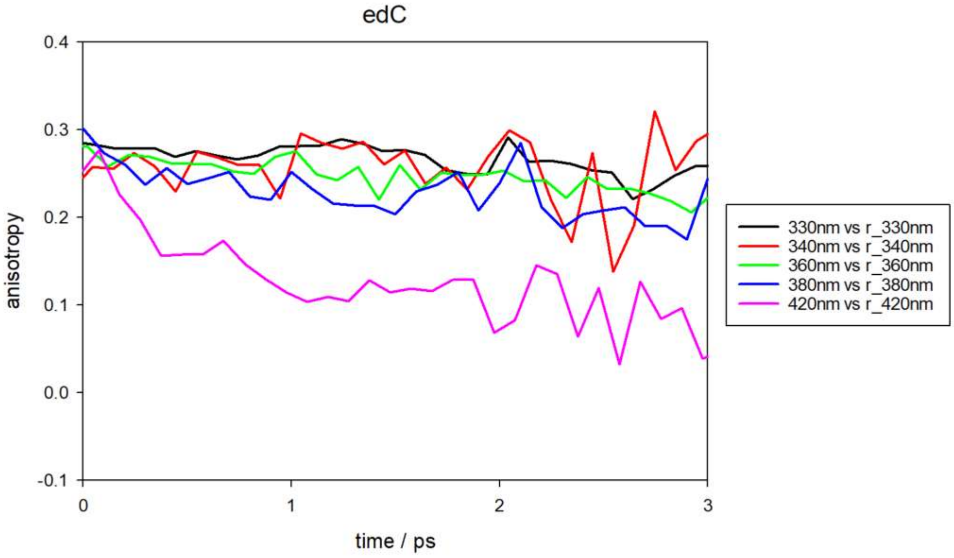

Figure S7. Selected anisotropy decays obtained from a solution of εdC in PBS pH 7.4 at different emission wavelengths after excitation at λ_{exc}= 267 nm.

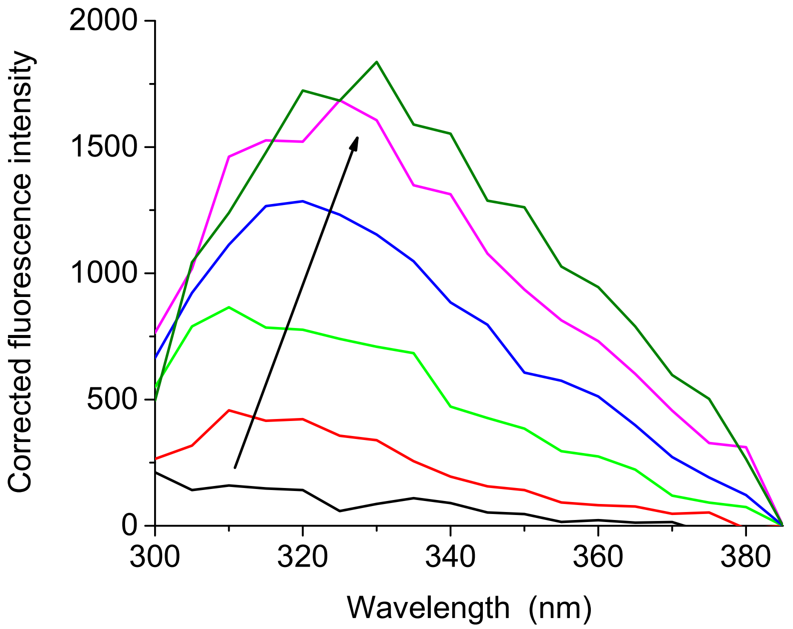

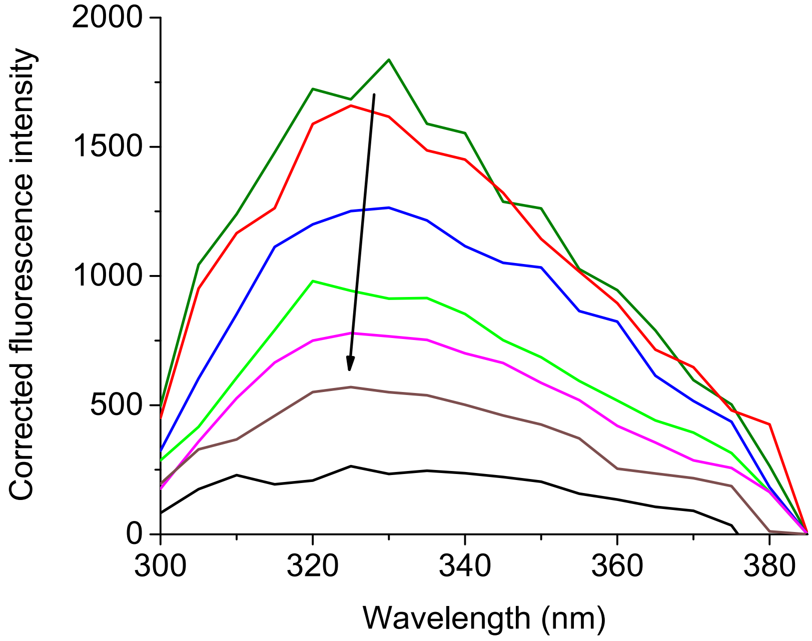

Figure S8. Fluorescence emission of εdC at pH 7.4 at different delays after the pump. (A) up to 0.3 ps, (B) from 0.3 to 2.7 ps

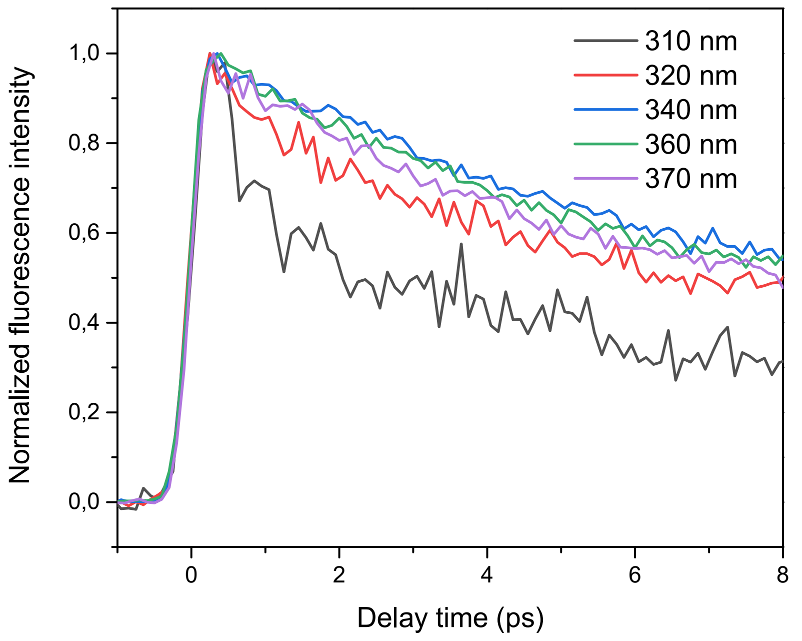

Figure S9. Total fluorescence decays, in semi-logarithmic scale, obtained for εdCH^{+} in PBS pH 3 at different emission wavelengths after excitation at λ_{exc}= 267 nm.

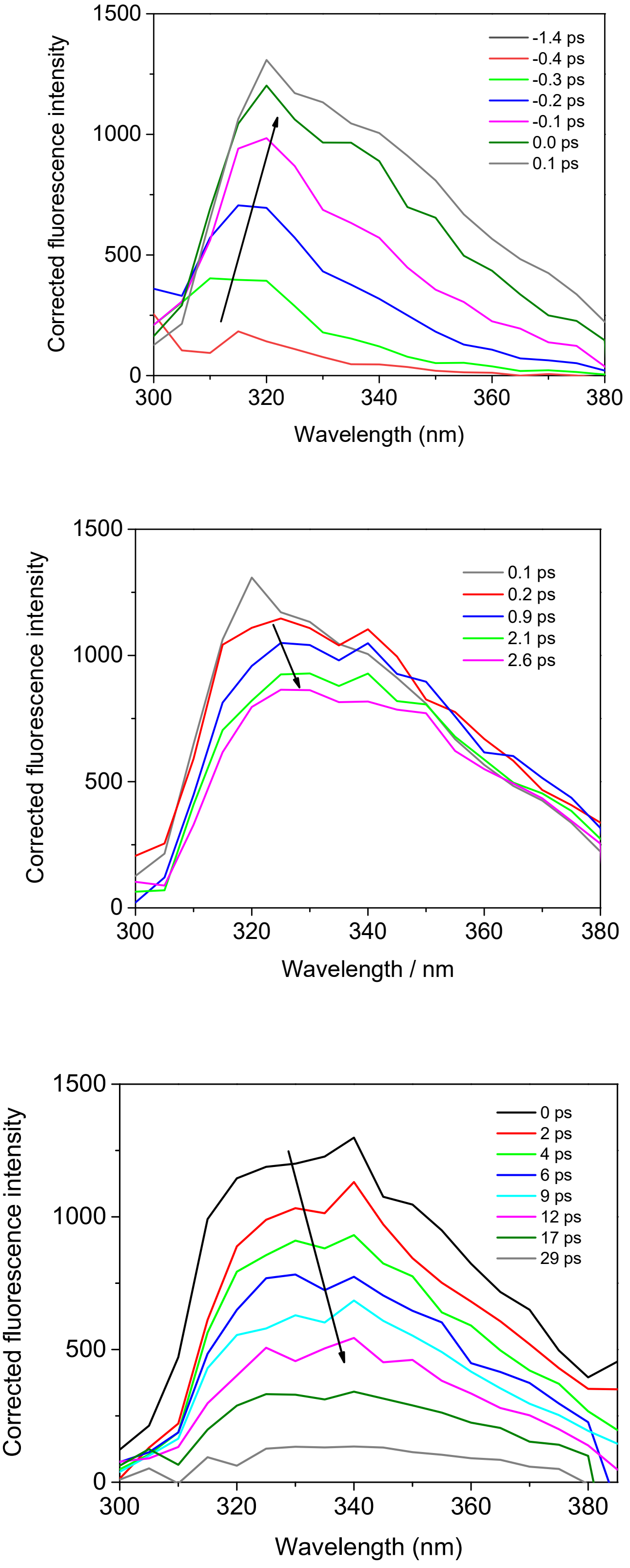

Figure S10. Fluorescence emission of εdCH$^+$ at pH 3 at different delays after the pump. (A) up to 0.1 ps, (B) from 0.1 to 2.6 ps, (C) at longer timescale from 0 to 29 ps

Lognorm Fitting Section:

For the spectral fitting of time-resolved fluorescence spectra, they were first converted from wavelength to wavenumber by multiplying with λ^2.

In order not to impose any specific time-dependence on the spectral parameters, each corrected time-resolved fluorescence spectrum was independently fitted with a simplified $\log_{norm}$ function[18]

$$I(\nu) = I_0 e^{\left(-\beta^2\left[ln\frac{\nu-\nu_0}{\Delta\nu}\right]^2\right)}$$

The peak frequency ν_P and the spectral with *fwhm* were calculated using the relations

$\nu_P = -\nu_0 - \Delta\nu$

fwhm = $2\Delta\nu sinh\left(\frac{\sqrt{ln}}{4\beta^2}\right)$.

Such a treatment provides information about the time-evolution of the intensity, the peak frequency and the spectral width. Below are shown the resulting curves for pH 7. The corresponding fitted lognormal functions are also shown as black solid lines.

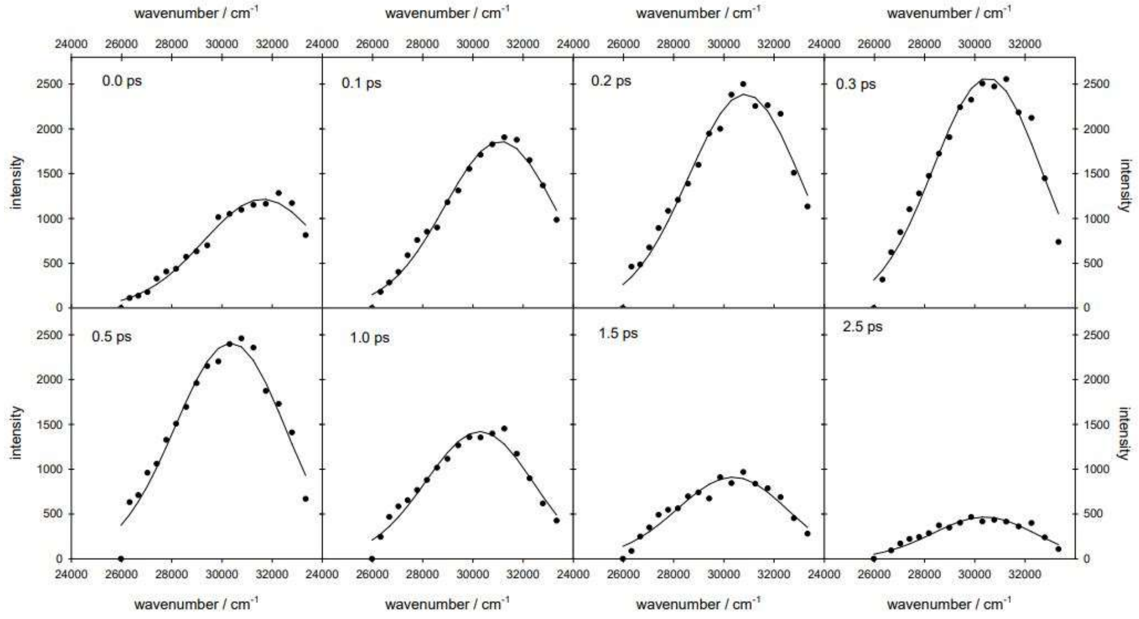

Figure S11. Selected time-resolved fluorescence spectra of εdC in PBS at pH 7.4 (black dots) at different delay times after excitation at λ_{exc}= 267 nm.

It is important to note that the time-resolved fluorescence spectra are not deconvoluted, so the rise in intensity is limited by the instrument response function (irf, 400 fs fwhm), in accordance with the fitting/deconvolution of the fluorescence decays.

The total intensity, peak frequency and spectral width as functions of time are shown in the figure below. It was found that the total intensity decays rapidly with a time constant of about 1 ps (Figure S12A), in accordance with the individual decays (average lifetime $<\tau>$, Table S5) It was found that the fluorescence spectrum undergoes an ultrafast red shift followed by a small amplitude and much slower blue shift stabilizing at 30400 cm^{-1} (Figure S8 and S12B). The former might correspond to water solvation dynamics occurring at sub-ps to ps timescale,[19,20] whereas the latter might be interpreted in terms of rearrangement of the sugar conformation as previously proposed for guanosine derivatives.[20] A ~ 10% decrease of the spectral width (Figure S12C) occurring within 3

ps, may correspond to the common signature of a vibrational cooling. In this context, previous studies proposed that the strong hydrogen bonding of the nucleoside to water molecules accelerates the cooling through an efficient intermolecular vibrational energy transfer to the solvent.[21]

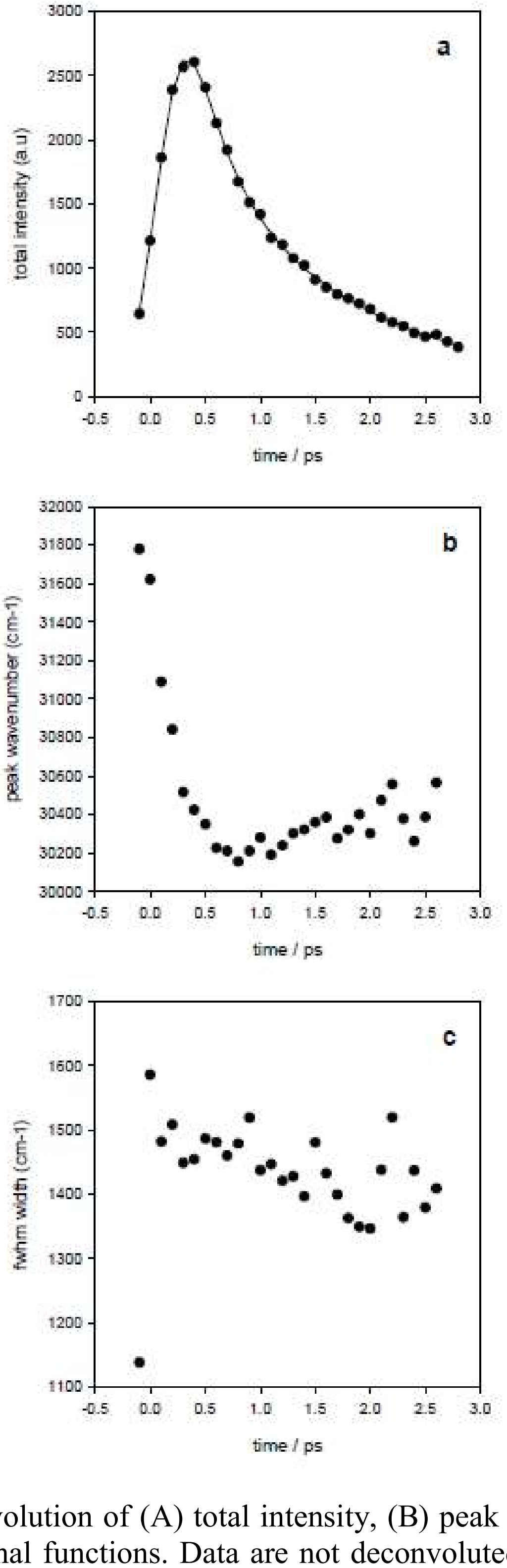

z

Figure S12. Time evolution of (A) total intensity, (B) peak frequency and (C) spectral width using log-normal functions. Data are not deconvoluted so the rise in intensity is only limited by the irf, in accordance with the fitting/deconvolution of the decays, giving a temporal width of about 400 fs.

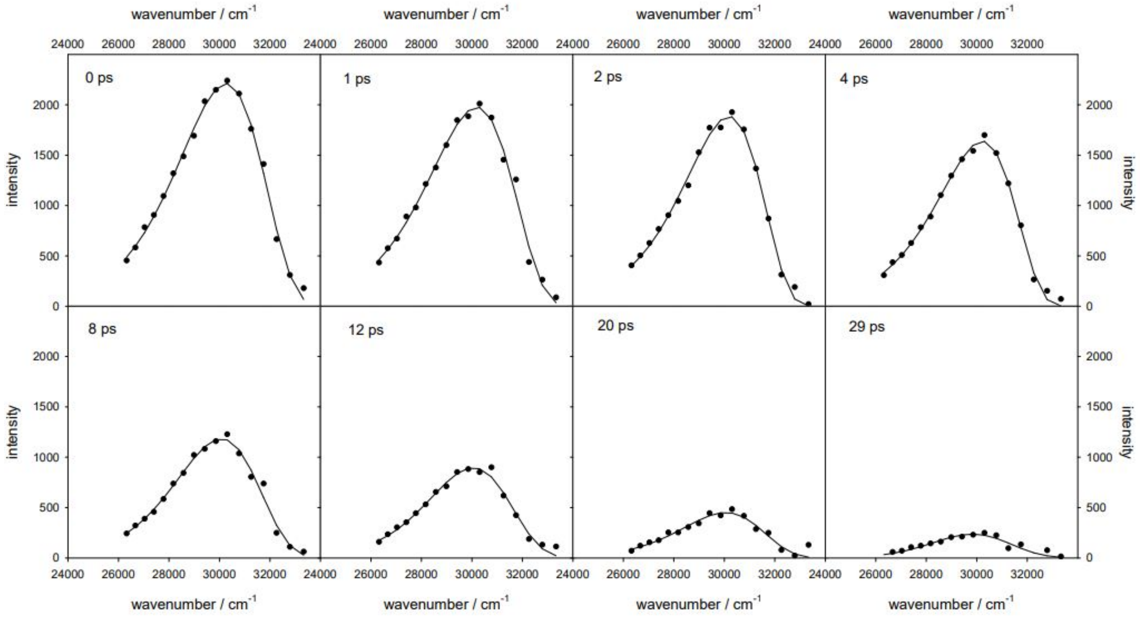

Figure S13. Selected time-resolved fluorescence spectra of εdC in PBS pH 3 (black dots) at different delay times after excitation at λ_{exc}= 267 nm. The corresponding fitted lognormal functions are also shown as black solid lines.

The total intensity, peak frequency and spectral width as functions of time are shown in the figure below. This analysis was performed for the two time-windows of 3 and 30 ps (Figure S13-S15). As in the case of pH 7.4, an ultrafast red shift can be observed (Figures S14B and S15B); then, beyond 1 ps, this shift proceeds more slowly all the spectra up to 30 ps (Figure S14B and S15B). Regarding the spectral width, an ultrafast broadening appears, once again limited by the 400 fs irf (Figure S14C), followed by a ps narrowing from ≈1350 cm^{-1} to ≈1300 cm^{-1}, caused by vibrational cooling of a 'hot' excited state population by energy dissipation to the surrounding solvent molecules.[22] At longer times, there is no visible change in the spectral width (Figure S15C).

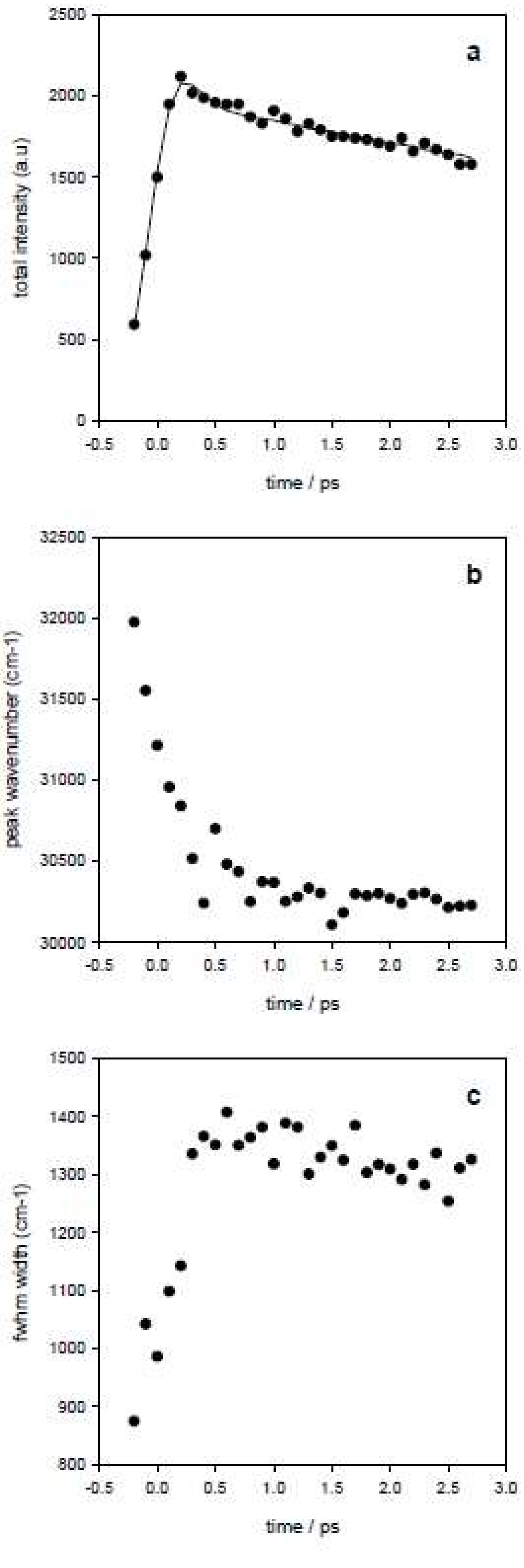

Figure S14. Time evolution of (A) total intensity, (B) peak frequency and (C) spectral width using log-normal functions over a time window of 3 ps. Data are not deconvoluted so the rise in intensity is only limited by the irf, in accordance with the fitting/deconvolution of the decays, giving a temporal width of about 400 fs.

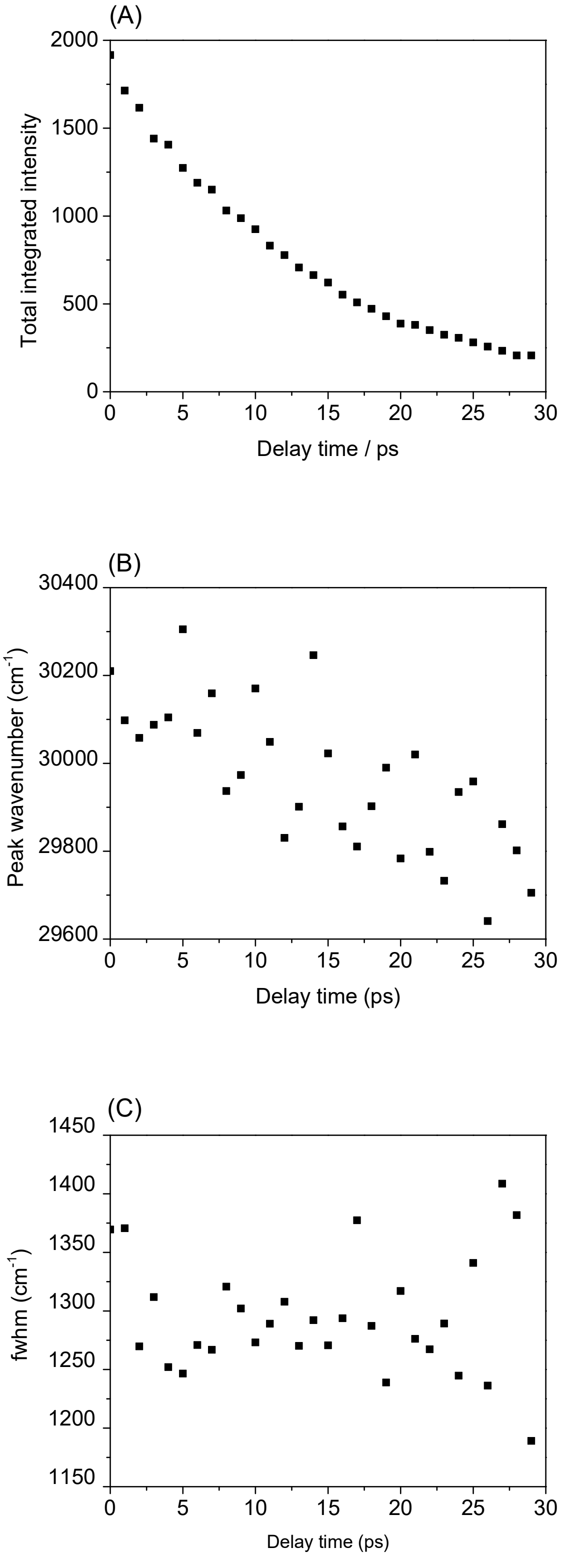

Figure S15. Time evolution of (A) total intensity, (B) peak frequency and (C) spectral width using log-normal functions over a time window of 30 ps.

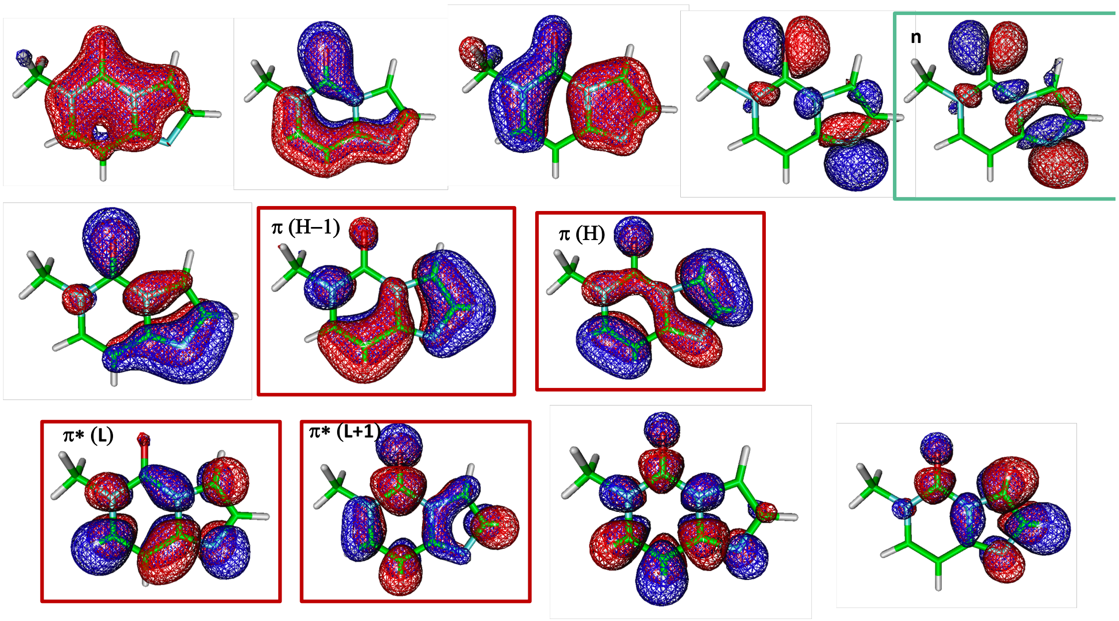

Figure S16. CASSCF Active Space for εC calculations. Framed in red the MO involves in ππ* excitations (ππ*1: H->L and ππ*2: H->L+1 + H-1->L) and in green the nπ* states (nπ*1: n->L and nπ*2:n->L+1).

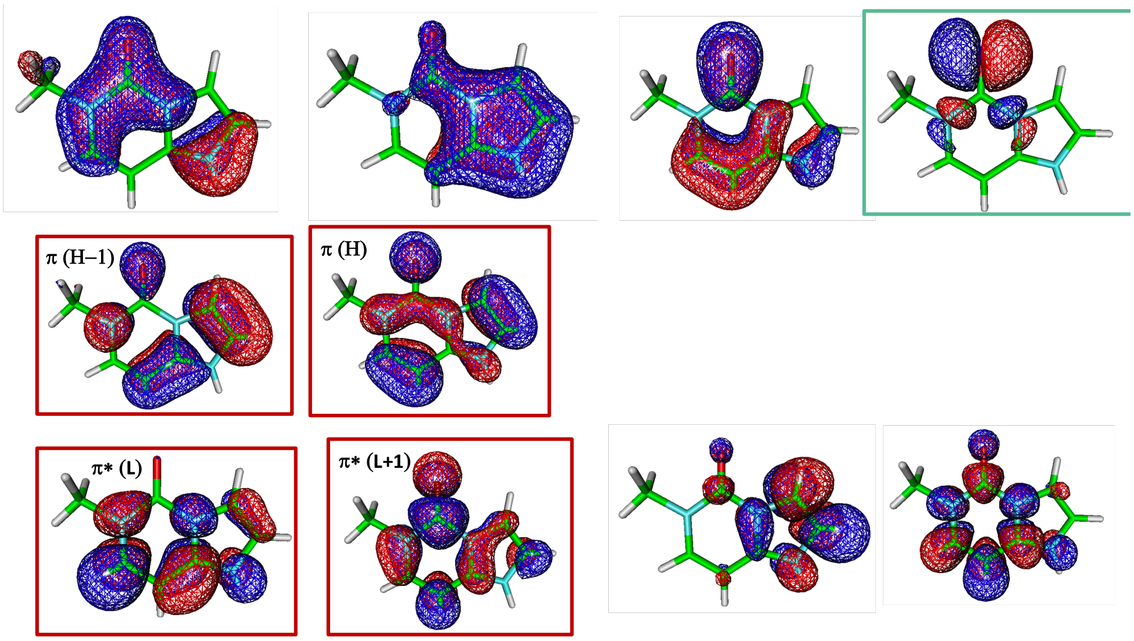

Figure S17. CASSCF Active Space for εCH^{+} calculations. Framed in red the MO involves in ππ* excitations (ππ*1: H->L and ππ*2: H->L+1 + H-1->L) and in green the nπ* states (nπ*1: n->L+1 and nπ*2:n->L).

Table S1. Optical properties of εdC and dC in PBS 0.1 M at pH 7.4 and 3, absorption maximum (λ_{max}) and its corresponding extinction coefficient (ε_{max}), steady-state emission fluorescence maximum (λ_{em}) and fluorescence quantum yield (ϕ_F) determined at λ_{exc}= 267 nm.

	λ_{max}	λ_{em}	ϕ_F	**ε ($M^{-1}cm^{-1}$)**
**εdC (pH 7.4)**	273 nm (4.54 eV)	325 nm (3.81 eV)	2.0 (± 0.1) $\times \cdot 10^{-4}$	13200
**εdCH^{+} (pH 3)**	284 nm (4.36 eV)	332 nm (3.7 eV)	3.2 (± 0.3) $\times \cdot 10^{-3}$	7200
**dC**[a]	271 nm	323 nm	$0.6 \cdot 10^{-4}$	9300

[a]in water, data from ref [5]

Table S2 Vertical absorption and emission energies computed for εC•2H_2O and εC in water. PCM/M052X/6-31G(d) geometry optimization. Oscillator strength is given in parentheses. The results have been obtained at the solvent non equilibrium level.

	**εC•2H_2O**		**εC**		**C**	
	**6-31G(d)**	**6-311+G(2d,2p)**	**6-31G(d)**	**6-311+G(2d,2p)**	**6-31G(d)**	**6-311+G(2d,2p)**
**S_1 (ππ*1)**	5.16(0.29)	4.99(0.31)	5.15(0.30)	4.98(0.32)	5.29(0.17)	5.13(0.25)
**S_1 (ππ*1-min) emission**	4.34 (0.35)	-	4.34 (0.35)	-	-	-
**S_1 (ππ*1-min) emission[a]**	4.17(0.49)[a]		4.16(0.49)[a]			
**S_2 (ππ*2)**	5.52 (0.11)	5.30(0.13)	5.56(0.10)	5.34(0.12)	6.27(0.22)[b]	6.06(0.17)
**S_3 (nπ*)**	6.42 (0.00)	6.26(0.04)	6.26(0.00)	6.17(0.04)	5.84(0.00)	5.87(0.00)

[a] emission energies computed at the solvent equilibrium level, [b]S_4

Table S3. Vertical absorption and emission energies computed for $\varepsilon CH^+ \bullet 2H_2O$ and εCH^+ in water. PCM/M052X/6-31G(d) calculations. Oscillator strength is given in parentheses. The results have been obtained at the solvent non equilibrium level.

	**$\varepsilon CH^+ \bullet 2H_2O$**		**εCH^+**	
	**6-31G(d)**	**6-311+G(2d,2p)**	**6-31G(d)**	**6-311+G(2d,2p)**
**S_1 (ππ*1)**	4.95(0.33)	4.84(0.34)	4.88(0.34)	4.78(0.35)
**S_1 (ππ*1-min) emission**	4.27(0.36)		4.24(0.36)	
**S_1 (ππ*1-min) emission[a]**	4.11(0.49)[a]		4.07(0.48)[a]	
**S_2 (ππ*2)**	5.82(0.15)	5.59(0.17)	5.93(0.14)	5.68(0.17)
**S_3 (nπ*)**	6.33(0.00)	6.39(0.00)	6.19(0.00)	6.25(0.00)

[a]emission energies computed at the solvent equilibrium level

Table S4. Vertical absorption and emission energies (in eV) computed for εC and εCH^+ in the gas phase. SS (italics) and MS-CASPT2 calculations. Oscillator strength is given in parentheses.

	**εC**		**εCH^+**	
	**character**		**character**	
**S1**	**ππ*1**	4.56(0.16) *4.88*	**ππ*1**	4.25(0.30) *4.25*
	**S_1 (ππ*1-min)**	3.52 (0.43) *4.17*	**S_1 (ππ*1-min)**	3.23 (0.18) *3.49*
**S2**	**ππ*2**	5.42(0.01)	**ππ*2**	5.73(0.02)
**S3**	**nπ*1**	5.92(0.01)	**nπ*1**	6.13(0.00)
**S4**	**nπ*2**	6.28(0.00)	**nπ*2**	6.24(0.00)

Table S5. Fitted parameters for the decays of εdC in PBS. The average lifetime was calculated as <τ> = $a_1\tau_1 + a_2\tau_2$. The anisotropy decay time $\tau_{R,2}$ was fixed to 55 ps.

λ (nm)	310 nm	320 nm	330 nm	340 nm	360 nm	380 nm	420 nm
a_1	0.585 ± 0.017	0.592 ± 0.010	0.550 ± 0.017	0.568 ± 0.031	0.658 ± 0.017	0.642 ± 0.017	0.618 ± 0.028
τ_1	0.260 ± 0.032	0.395 ± 0.0198	0.455 ± 0.028	0.479 ± 0.044	0.547 ± 0.021	0.512 ± 0.023	0.537 ± 0.046
p_1[a]	0.178 ± 0.021	0.237 ± 0.012	0.226 ± 0.017	0.260 ± 0.032	0.341 ± 0.020	0.313 ± 0.020	0.268 ± 0.031
a_2	0.415 ± 0.017	0.408 ± 0.010	0.450 ± 0.018	0.431 ± 0.031	0.342 ± 0.017	0.358 ± 0.017	0.382 ± 0.028
τ_2	1.686 ± 0.051	1.849 ± 0.037	1.899 ± 0.056	1.796 ± 0.084	2.029 ± 0.062	2.011 ± 0.061	2.378 ± 0.130
p_2[a]	0.822 ± 0.021	0.763 ± 0.012	0.774 ± 0.017	0.740 ± 0.032	0.659 ± 0.020	0.687 ± 0.020	0.732 ±0.031
<τ>	0.852 ± 0.037	0.988 ± 0.024	1.106 ± 0.039	1.048 ± 0.060	1.054 ± 0.036	1.049 ± 0.036	1.240 ± 0.077
r_0	0.296 ± 0.005	0.285 ± 0.002	0.278 ± 0.002	0.273 ± 0.004	0.261 ± 0.002	0.270 ± 0.007	0.275 ± 0.014
a_r	(1)	(1)	(1)	(1)	(1)	0.178 ± 0.025	0.626 ± 0.031
$\tau_{R,1}$						0.437 ± 0.198	0.348 ± 0.076
$\tau_{R,2}$	(55)	(55)	(55)	(55)	(55)	(55)	(55)

[a] relative contributions p_1 and p_2 have been calculated as follows: $p_1 = a_1\tau_1/(a_1\tau_1+a_2\tau_2)$ and $p_2 = a_2\tau_2/(a_1\tau_1+a_2\tau_2)$, [b]The average lifetime was calculated as <τ> = $a_1\tau_1 + a_2\tau_2$, [c]fixed value.

Table S6. Fitted parameters for the decays of εdCH$^+$ at pH 3.

λ (nm)	310 nm	320 nm	340 nm	360 nm	370 nm
**a_1**	0.557 ± 0.020	0.213 ± 0.012	0.128 ± 0.089	0.139 ± 0.011	0.183 ± 0.007
**$τ_1$ (ps)**	0.339 ± 0.045	0.676 ± 0.083	0.124 ± 0.074	1.70 ± 0.23	1.91 ± 0.18
**p_1**	0.039 ± 0.006	0.014 ± 0.002	0.0014 ± 0.0014	0.019 ± 0.003	0.028 ± 0.003
**a_2**	0.443 ± 0.020	0.787 ± 0.012	0.872 ± 0.089	0.861 ± 0.011	0.817 ± 0.007
**$τ_2$ (ps)**	10.51 ± 0.53	12.53 ± 0.24	12.72 ± 0.09	14.15 ±- 0.35	(15)
**p_2**	0.961 ± 0.006	0.986 ± 0.002	0.99986 ± 0.0014	0.981 ± 0003	0.972 ± 0.003
**<τ> (ps)**	4.85 ± 0.31	10.01 ± 0.24	11.10 ± 1.12	12.42 ± 0.33	12.61 ± 0.09
**r_0**	0.323 ± 0.007	0.325 ± 0.003	0.320 ± 0.002	0.314 ± 0.002	0.315 ± 0.002
**$τ_R$ (ps)**	(55)	(55)	(55)	(55)	(55)

References:

(1) Onidas, D.; Markovitsi, D.; Marguet, S.; Sharonov, A.; Gustavsson, T. Fluorescence Properties of DNA Nucleosides and Nucleotides: A Refined Steady-State and Femtosecond Investigation. *J. Phys. Chem. B* **2002**, *106*, 11367–11374. DOI: 10.1021/jp026063g.

(2) Miannay, F. A.; Gustavsson, T.; Banyasz, A.; Markovitsi, D. Excited-State Dynamics of DGMP Measured by Steady-State and Femtosecond Fluorescence Spectroscopy. *J. Phys. Chem. A* **2010**, *114*, 3256–3263. DOI: 10.1021/jp909410b.

(3) Gustavsson, T.; Bányász, Á.; Lazzarotto, E.; Markovitsi, D.; Scalmani, G.; Frisch, M. J.; Barone, V.; Improta, R. Singlet Excited-State Behavior of Uracil and Thymine in Aqueous Solution: A Combined Experimental and Computational Study of 11 Uracil Derivatives. *J. Am. Chem. Soc.* **2006**, *128*, 607–619. DOI: 10.1021/ja056181s.

(4) Tomasi, J.; Mennucci, B.; Cammi, R. Quantum Mechanical Continuum Solvation Models. *Chem. Rev.* **2005**, *105*, 2999–3093. DOI: 10.1021/cr9904009.

(5) Martínez-Fernández, L.; Pepino, A. J.; Segarra-Martí, J.; Jovaišaitei, J.; Vaya, I.; Nenov, A.; Markovitsi, D.; Gustavsson, T.; Banyasz, A.; Garavelli, M.; Improta, R. Photophysics of Deoxycytidine and 5-Methyldeoxycytidine in Solution: A Comprehensive Picture by Quantum Mechanical Calculations and Femtosecond Fluorescence Spectroscopy. *J. Am. Chem. Soc.* **2017**, *139*, 7780–7791. DOI: 10.1021/jacs.7b01145.

(6) Zhao, Y.; Schultz, N. E.; Truhlar, D. G. Design of Density Functionals by Combining the Method of Constraint Satisfaction with Parametrization for Thermochemistry, Thermochemical Kinetics, and Noncovalent Interactions. *J. Chem. Theory Comput.* **2006**, *2*, 364–382. DOI: 10.1021/ct0502763.

(7) Zhao, Y.; Truhlar, D. G. Density Functionals with Broad Applicability in Chemistry. *Acc. Chem. Res.* **2008**, *41*, 157–167. DOI: 10.1021/ar700111a.

(8) Improta, R.; Santoro, F.; Blancafort, L. Quantum Mechanical Studies on the Photophysics and the Photochemistry of Nucleic Acids and Nucleobases. *Chem. Rev.* **2016**, 116, 3540–3593. DOI: 10.1021/acs.chemrev.5b00444.

(9) Frisch, M. J.; Trucks, G. W.; Schlegel, H. B.; Scuseria, G. E.; Robb, M. A.; Cheeseman, J. R.; Scalmani, G.; Barone, V.; Petersson, G. A.; Nakatsuji, H.; Li, X.; Caricato, M.; Marenich, A. V.; Bloino, J.; Janesko, B. G.; Gomperts, R.; Mennucci, B.; Hratchian, H. P.; Ortiz, J. V.; Izmaylov, A. F.; Sonnenberg, J. L.; Williams-Young, D.; Ding, F.; Lipparini, F.; Egidi, F.; Goings, J.; Peng, B.; Petrone, A.; Henderson, T.; Ranasinghe, D.; Zakrzewski, V. G.; Gao, J.; Rega, N.; Zheng, G.; Liang, W.; Hada, M.; Ehara, M.; Toyota, K.; Fukuda, R.; Hasegawa, J.; Ishida, M.; Nakajima, T.; Honda, Y.; Kitao, O.; Nakai, H.; Vreven, T.; Throssell, K.; Montgomery, J. A., J.; Peralta, J. E.; Ogliaro, F.; Bearpark, M. J.; Heyd, J. J.; Brothers, E. N.; Kudin, K. N.; Staroverov, V. N.; Keith, T. A.; Kobayashi, R.; Normand, J.; Raghavachari, K.; Rendell, A. P.; Burant, J. C.; Iyengar, S. S.; Tomasi, J.; Cossi, M.; Millam, J. M.; Klene, M.; Adamo, C.; Cammi, R.; Ochterski, J. W.; Martin, R. L.; Morokuma, K.; Farkas, O.; Foresman, J. B.; Fox, D. J. Gaussian 16 Revision C01. 2016, p Gaussian 16, Revision C.01, Gaussian, Inc., Wallin.

(10) Andersson, K.; Malmqvist, P. A.; Roos, B. O.; Sadlej, A. J.; Wolinski, K. Second-Order Perturbation Theory with a CASSCF Reference Function. *J. Phys. Chem.* **1990**, *94*, 5483–5488. DOI: 10.1021/j100377a012.

(11) Andersson, K.; Malmqvist, P.; Roos, B. O. Second-order Perturbation Theory with a Complete Active Space Self-consistent Field Reference Function. *J. Chem. Phys.* **1992**, *96*, 1218–1226. DOI: 10.1063/1.462209.

(12) Finley, J.; Malmqvist, P.-Å.; Roos, B. O.; Serrano-Andrés, L. The Multi-State CASPT2 Method. *Chem. Phys. Lett.* **1998**, *288*, 299–306. DOI: 10.1016/S0009-2614(98)00252-8.

(13) Ghigo, G.; Roos, B. O.; Malmqvist, P.-Å. A Modified Definition of the Zeroth-Order Hamiltonian in

Multiconfigurational Perturbation Theory (CASPT2). *Chem. Phys. Lett.* **2004**, *396*, 142–149. DOI: 10.1016/j.cplett.2004.08.032.

(14) Forsberg, N.; Malmqvist, P.-Å. Multiconfiguration Perturbation Theory with Imaginary Level Shift. *Chem. Phys. Lett.* **1997**, *274*, 196–204. DOI: 10.1016/S0009-2614(97)00669-6.

(15) Fernández Galván, I.; Vacher, M.; Alavi, A.; Angeli, C.; Aquilante, F.; Autschbach, J.; Bao, J. J.; Bokarev, S. I.; Bogdanov, N. A.; Carlson, R. K.; Chibotaru, L. F.; Creutzberg, J.; Dattani, N.; Delcey, M. G.; Dong, S. S.; Dreuw, A.; Freitag, L.; Frutos, L. M.; Gagliardi, L.; Gendron, F.; Giussani, A.; Gonzalez, L.; Grell, G.; Guo, M.; Hoyer, C. E.; Johansson, M.; Keller, S.; Knecht, S.; Kovačević, G.; Källman, E.; Li Manni, G.; Lundberg, M.; Ma, Y.; Mai, S.; Malhado, J. P.; Malmqvist, P. A.; Marquetand, P.; Mewes, S. A.; Norell, J.; Olivucci, M.; Oppel, M.; Phung, Q. M.; Pierloot, K.; Plasser, F.; Reiher, M.; Sand, A. M.; Schapiro, I.; Sharma, P.; Stein, C. J.; Sørensen, L. K.; Truhlar, D. G.; Ugandi, M.; Ungur, L.; Valentini, A.; Vancoillie, S.; Veryazov, V.; Weser, O.; Wesolowski, T. A.; Widmark, P.-O.; Wouters, S.; Zech, A.; Zobel, J. P.; Lindh, R. OpenMolcas: From Source Code to Insight. *J. Chem. Theory Comput.* **2019**, *15*, 5925–5964. DOI: 10.1021/acs.jctc.9b00532.

(16) Martin, R. L. Natural Transition Orbitals. *J. Chem. Phys.* **2003**, *118*, 4775–4777. DOI: 10.1063/1.1558471.

(17) Avila Ferrer, F. J.; Cerezo, J.; Stendardo, E.; Improta, R.; Santoro, F. Insights for an Accurate Comparison of Computational Data to Experimental Absorption and Emission Spectra: Beyond the Vertical Transition Approximation. *J. Chem. Theory Comput.* **2013**, *9*, 2072–2082. DOI: 10.1021/ct301107m.

(18) Gustavsson, T.; Cassara, L.; Gulbinas, V.; Gurzadyan, G.; Mialocq, J. C.; Pommeret, S.; Sorgius, M.; Van Der Meulen, P. Femtosecond Spectroscopic Study of Relaxation Processes of Three Amino-Substituted Coumarin Dyes in Methanol and Dimethyl Sulfoxide. *J. Phys. Chem. A* **1998**, *102*, 4229–4245. DOI: 10.1021/jp980282d.

(19) Jimenez, R.; Fleming, G. R.; Kumar, P. V; Maroncelli, M. Femtosecond Solvation Dynamics of Water. *Nature* **1994**, *369*, 471–473. DOI: 10.1038/369471a0.

(20) Martinez-Fernandez, L.; Gustavsson, T.; Diederichsen, U.; Improta, R. Excited State Dynamics of 8-Vinyldeoxyguanosine in Aqueous Solution Studied by Time-Resolved Fluorescence Spectroscopy and Quantum Mechanical Calculations. *Molecules*. **2020**, *25*, 824. DOI: 10.3390/molecules25040824.

(21) Pecourt, J. M. L.; Peon, J.; Kohler, B. DNA Excited-State Dynamics: Ultrafast Internal Conversion and Vibrational Cooling in a Series of Nucleosides. *J. Am. Chem. Soc.* **2001**, *123*, 10370–10378. DOI: 10.1021/ja0161453.

(22) Gustavsson, T.; Baldacchino, G.; Mialocq, J.-C.; Pommeret, S. A Femtosecond Fluorescence Up-Conversion Study of the Dynamic Stokes Shift of the DCM Dye Molecule in Polar and Non-Polar Solvents. *Chem. Phys. Lett.* **1995**, *236*, 587–594. DOI: 10.1016/0009-2614(95)00276-A.
